# Association Between Glycated Hemoglobin and Coronary Artery Calcification in Middle-Aged and Elderly Chinese Checkup Populations

**DOI:** 10.5812/ijem-158710

**Published:** 2025-04-21

**Authors:** Ya Huang, Wenji Ni, Ying Zhou, Dandan Li, Rui Zhang, Tao Jin, Yong Zhong

**Affiliations:** 1 Department of Health Medicine, Jinling Hospital, Affiliated Hospital of Medical School, Nanjing University, Nanjing, China

**Keywords:** Glycated Hemoglobin, Coronary Artery Calcification, Computed Tomography, Cardiovascular Disease, Atherosclerosis

## Abstract

**Background:**

Previous studies have established that coronary artery calcification (CAC) is a robust predictor of adverse cardiovascular events.

**Objectives:**

To examine the association between levels of glycated hemoglobin (HbA1c), an indicator of long-term blood glucose levels, and CAC in middle-aged and elderly Chinese populations undergoing routine health screenings.

**Methods:**

A cross-sectional study was conducted on 8,955 Chinese adults over 40 years of age who underwent physical examinations in the Department of Health Medicine at our hospital from January 2022 to July 2023. The odds ratios (ORs) of CAC in relation to HbA1c were determined using multiple logistic regression analysis, both as a continuous and categorical variable. Furthermore, dose-response relationships between HbA1c levels and CAC were visualized using restricted cubic spline models.

**Results:**

Compared to the group with HbA1c lower than 5.7%, individuals in the groups with HbA1c of 5.7% to 6.4% and ≥ 6.5% exhibited an elevated prevalence of CAC (P for trend < 0.0001). Multivariable logistic regression showed that each 1% increase in HbA1c was associated with a 24% increased risk of CAC (OR = 1.24, 95% CI: 1.03-1.48, P = 0.02). Compared with the group with HbA1c lower than 5.7%, the groups with HbA1c at 5.7% - 6.4% and HbA1c ≥ 6.5% were associated with a 28% (OR = 1.28, 95% CI: 1.07 - 1.52) and 116% (OR = 2.16, 95% CI: 1.48 - 3.16) (P for trend < 0.0001) increased risk of CAC, respectively. Restricted cubic spline analyses showed a non-linear association between HbA1c and CAC (P for nonlinearity < 0.0001). At higher levels of HbA1c exposure (> 5.7%), the exposure dose-response curves appeared upward-sloping. Subgroup analyses showed that the association between HbA1c and CAC was more pronounced in those aged less than 60 years, with normal weight and blood pressure less than 135/85 mmHg, although none of the interactions between HbA1c and subgroups were statistically significant.

**Conclusions:**

This study indicated that higher HbA1c levels are associated with a greater likelihood of CAC in the middle-aged and elderly Chinese checkup population.

## 1. Background

Atherosclerosis is a significant factor in the high rates of morbidity and mortality worldwide, with millions of deaths occurring annually due to cardiovascular disease (1). Approximately 330 million people in China alone are living with cardiovascular disease, and the incidence of this condition is on the rise, presenting a major public health challenge (2). Atherosclerosis is the primary cause of conditions such as ischemic heart disease, making early detection of subclinical atherosclerosis essential for prevention. Coronary artery calcification (CAC), linked to advanced atherosclerosis, is crucial for evaluating the presence and severity of coronary artery disease (3). The advent of multislice computed tomography (CT) has enabled the precise identification of CAC, offering crucial details on the overall burden of atherosclerotic plaque (4). Previous research has indicated that individuals with diabetes are more likely to have subclinical coronary atherosclerosis, as measured by CAC (5). According to research from the heinz nixdorf recall study, individuals with prediabetes may have a higher prevalence of CAC (6), suggesting that moderate dysglycemia could potentially initiate the onset of CAC. Early detection and treatment of dysglycemia are essential to prevent the onset of coronary artery disease. Fasting plasma glucose, 2-hour postprandial glucose, and glycated hemoglobin (HbA1c) are all critical indicators of glycemic abnormalities. However, HbA1c is considered a more reliable biomarker than fasting or postprandial glucose, as it provides a more accurate and comprehensive reflection of long-term glycemic control (7). Previous research has indicated that increased HbA1c levels pose a risk for cardiovascular disease in both diabetic and non-diabetic populations (8). A study conducted by Chang et al. discovered that elevated HbA1c levels were independently linked to subclinical coronary atherosclerosis in non-diabetic women (9). Moreover, Won et al. (as cited by Kowall) showed that maintaining optimal glycemic control (HbA1c < 7.0%) can delay the progression of CAC in asymptomatic diabetic patients (10). However, there is insufficient data regarding the association between HbA1c levels and CAC in the overall population.

## 2. Objectives

In this study, we explored the association between CAC and rising levels of HbA1c among a Chinese health checkup group. Additionally, we investigated whether this association remains unaffected by conventional cardiovascular risk factors and if it holds true across various population subgroups.

## 3. Methods

### 3.1. Study Design and Population

The research was conducted as a cross-sectional study, involving 9,052 individuals who underwent physical examinations with complete health check-up data at Jinling Hospital, Affiliated Hospital of Medical School, Nanjing University, from January 2022 to July 2023. The participants came from various institutions in Jiangsu province and had diverse socioeconomic backgrounds. Participation in the health checkups was voluntary but encouraged by employers and provided free of charge. During the examinations, participants completed a standard questionnaire on lifestyle factors, medical history, and diseases. Anthropometric measurements and blood samples were taken by trained nurses. Individuals with chronic heart failure, renal failure, liver failure, severe anemia, hematological diseases, malignant tumors (n = 58), and those with coronary heart disease who had undergone coronary artery stent implantation surgery (n = 39) were excluded from the study, leaving 8,955 eligible participants.

### 3.2. Data Collection

Trained doctors gathered detailed information about age, gender, smoking habits, alcohol consumption, medical history, and medication usage through questioning. Height and weight were measured using the SH-200G instrument. Body Mass Index (BMI) was calculated by dividing weight in kilograms by height in meters squared. Blood pressure was taken on the non-dominant arm after a 10-minute rest using an Omron blood pressure monitor. Venous blood samples were collected from fasting subjects (fasting for a minimum of eight hours). Levels of fasting blood glucose (FBG), triglycerides (TG), total cholesterol (TC), high-density lipoprotein cholesterol (HDL-C), low-density lipoprotein cholesterol (LDL-C), serum creatinine (SCR), and uric acid (UA) were measured using a HITACHI 7600 automatic biochemical analyzer. Postprandial blood glucose was measured 2 hours after breakfast. The HbA1c levels were determined using ion-exchange high-pressure liquid chromatography. Laboratory test quality control was conducted in compliance with the "Medical Quality Control Indicators for Clinical Laboratory Specialties" (2015 edition) set forth by the National Health Commission of the People's Republic of China. Accuracy was evaluated through bias, while precision was assessed using the coefficient of variation (CV). The specific values for the quality control of each laboratory indicator are presented in Appendix 1 in Supplementary File of the Supplementary Material. Estimated glomerular filtration rate (eGFR) was calculated using the modified MDRD equation: eGFR [mL/(min × 1.73 m^2^) = 186 × (SCR)^-1.154^ × (age)^-0.203 ^× (0.742 female) × (1.233 Chinese).

### 3.3. Assessment of Coronary Artery Calcification

**Figure 1.**
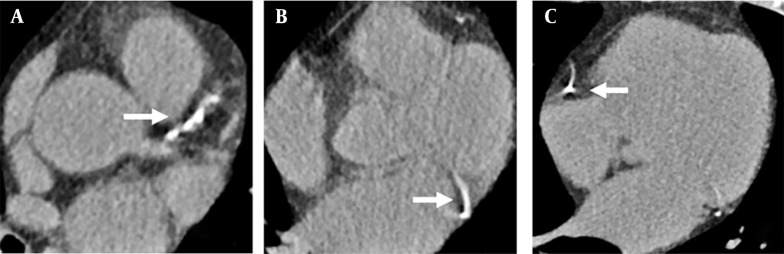
Overall visual assessment of coronary artery calcification (CAC); A, showed the calcification of the left anterior descending branch; B, showed the calcification of the circumflex branch; C, showed the calcification of the coronary artery. The white arrows represent calcification of the plate.

Each participant underwent a chest CT scan using a 64-slice multi-slice CT machine (Siemens somatom definition flash). The individual was positioned in a supine position, and the scan was conducted while holding their breath after taking a deep inhalation. The scanning parameters included a tube voltage of 120 kV, tube current ranging from 20 - 50 mA, a reconstruction layer thickness of 1 mm, and a layer distance of 1 mm. The scan covered the entire length from the top of the lungs to the bottom of the diaphragm, and the images were transferred to the imaging diagnostic workstation for storage. Two experienced radiologists visually assessed CAC on the chest CT images of the entire coronary circulation (11) (Figure 1). In cases where the two radiologists had differing opinions, a consensus was reached through discussion.

### 3.4. Statistical Analysis

We used SAS software, version 9.4 (SAS Institute, Cary, NC), for our statistical analysis. A two-sided P value of less than 0.05 was considered statistically significant. The HbA1c levels were categorized into three groups based on values of 5.6% and 6.5%: HbA1c < 5.7%, HbA1c at 5.7% - 6.4%, and HbA1c ≥ 6.5%. Normally distributed continuous variables were presented as mean ± standard deviation (SD), while non-normally distributed data were described as median (interquartile range) [M (Q1, Q3)]. Categorical variables were presented as counts and percentages. Linear regression for continuous variables and the Cochran-Armitage trend χ^2^ test for categorical variables were applied to analyze the trends across the three HbA1c groups. Logistic regression was used to calculate odds ratios (ORs) for the relationship between HbA1c levels and CAC in both overall and subgroup analyses. During subgroup analysis, interactions were analyzed by incorporating the product term between HbA1c and the subgroup (age, sex, BMI, and blood pressure) into the model. To allow for more flexibility in the model and visualize dose-response relationships, restricted cubic spline models with four knots at the 5th, 35th, 65th, and 95th percentiles of HbA1c were constructed.

## 4. Results

### 4.1. Characteristics of Study Participants

Among the 8,955 participants, 6,606 (73.77%) were men, with an average age of 50.05 years (SD 12.12) and an average HbA1c of 5.73% (SD 0.70%). A total of 1,700 individuals (18.98%) had CAC, including 1,507 men and 193 women. Table 1 shows the demographic and metabolic characteristics of the participants based on their HbA1c levels. Individuals with HbA1c levels between 5.7% and 6.4% and ≥ 6.5% had higher levels of age, male proportion, systolic blood pressure, diastolic blood pressure, TG, FBG, and 2-hour postprandial blood glucose compared to those with HbA1c levels lower than 5.7%. They also had lower levels of HDL-C and eGFR (all P for trend < 0.0001). There was no significant difference in TC, LDL-C, and UA levels among the three groups, with these levels being highest in the group with HbA1c levels between 5.7% and 6.4%. The number and percentage of individuals with CAC in the three HbA1c groups were 537 (11.53%), 825 (22.87%), and 338 (48.99%), respectively. The prevalence of CAC significantly increased with higher HbA1c levels (P for trend < 0.0001).

**Table 1.** Characteristics of Study Population According to Different Glycated Hemoglobin Status ^a^

Characteristics	HbA1c < 5.7 (n = 4658)	HbA1c 5.7 - 6.4 (n = 3607)	HbA1c ≥ 6.5 (n = 690)	P for Trend
**Age (y)**	46.12 ± 9.84	52.91 ± 11.78	61.64 ± 15.68	< 0.0001
**Gender (male)**	3201 (68.72)	2833 (78.54)	572 (82.90)	< 0.0001
**BMI (kg/m** ^**2**^ **)**	24.06 ± 3.48	24.85 ± 3.02	26.12 ± 3.58	< 0.0001
**Systolic blood pressure (mmHg)**	120.04 ± 14.73	124.03 ± 15.69	132.40 ± 17.66	< 0.0001
**Diastolic blood pressure (mmHg)**	73.78 ± 10.41	74.91 ± 10.53	75.99 ± 10.96	< 0.0001
**TC (mmol/L)**	5.04 ± 0.89	5.20 ± 1.02	4.95 ± 1.22	0.02
**TG (mmol/L)**	1.13 (0.79 - 1.67)	1.36 (0.95 - 1.96)	1.55 (1.09 - 2.28)	< 0.0001
**HDL - cholesterol (mmol/L)**	1.37 ± 0.34	1.36 ± 0.33	1.24 ± 0.31	< 0.0001
**LDL - cholesterol (mmol/L)**	2.75 ± 0.73	2.89 ± 0.80	2.71 ± 0.90	0.01
**Fasting plasma glucose (mmol/L)**	4.9 (4.7 - 5.2)	5.3 (5.0 - 5.7)	7.2 (6.3 - 8.6)	< 0.0001
**2h plasma glucose (mmol/L)**	5.7 (5.0 - 6.6)	6.4 (5.5 - 7.6)	11.5 (9.1 - 14.3)	< 0.0001
**UA (umol/L)**	355.76 ± 90.83	375.73 ± 88.55	357.43 ± 88.96	0.01
**eGFR (mL/min/1.73 m** ^**2**^ **)**	125.73 ± 22.96	121.49 ± 30.66	118.81 ± 22.64	< 0.0001
**HbA1c**	5.4 (5.2 - 5.5)	5.9 (5.7 - 6.0)	7.1 (6.7 - 7.8)	< 0.0001
**CAC**	537 (11.53)	825 (22.87)	338 (48.99)	< 0.0001

Abbreviations: eGFR, estimated glomerular filtration rate; CAC, coronary artery calcification; BMI, Body Mass Index; HbA1c, glycated hemoglobin; UA, uric acid; TG, triglyceride.

^a^ Values are expressed as means ± SD, medians (IQR) or No. (%).

### 4.2. Association of Glycated Hemoglobin with Risk of Coronary Artery Calcification

In Table 2, it is evident that for every 1% increase in HbA1c, there is a 38% higher risk of CAC after adjusting for age and sex (OR = 1.38, 95% CI: 1.27 - 1.49, P < 0.0001). The results remained consistent even after further adjustments for BMI, blood pressure, serum lipid, eGFR, and UA (OR = 1.33, 95% CI: 1.22 - 1.46, P < 0.0001). Additionally, when plasma glucose levels were taken into account, including fasting plasma glucose and 2-hour postprandial blood glucose, the association weakened slightly but remained statistically significant (OR = 1.24, 95% CI: 1.03 - 1.48, P = 0.02).

**Table 2.** The Association of Glycated Hemoglobin with Risk of Coronary Artery Calcification ^a^

Variables	Model 1		Model 2		Model 3	
	OR (95% CI)	P-Value	OR (95% CI)	P-Value	OR (95% CI)	P-Value
**Continuous**		< 0.0001		< 0.0001		0.02
HbA1c, per 1%	1.38 (1.27 - 1.49)		1.33 (1.22 - 1.46)		1.24 (1.03 - 1.48)	
**Categorical**		< 0.0001		< 0.0001		0.0001
HbA1c < 5.7%	Ref.		Ref.		Ref.	
HbA1c 5.7% - 6.4%	1.32 (1.16 - 1.51)		1.25 (1.08 - 1.45)		1.28 (1.07 - 1.52)	
HbA1c ≥ 6.5%	2.61 (2.13 - 3.19)		2.28 (1.79 - 2.91)		2.16 (1.48 - 3.16)	

Abbreviation: HbA1c, glycated hemoglobin; eGFR, estimated glomerular filtration rate.

^a^ Model 1, adjusted for age and sex; model 2, additionally adjusted for Body Mass Index, systolic blood pressure, diastolic blood pressure, total cholesterol, triglyceride, LDL-cholesterol, HDL-cholesterol, uric acid and eGFR based on model 1; model 3, additionally adjusted for fasting blood glucose and 2h plasma glucose bases on model 2.

The categorical analysis revealed similar findings. Compared to the group with HbA1c levels lower than 5.7%, those with HbA1c levels between 5.7% and 6.4% and those with HbA1c levels of 6.5% or higher exhibited a 32% (OR = 1.32, 95% CI: 1.16 - 1.51) and 161% (OR = 2.61, 95% CI: 2.13 - 3.19) increased risk of CAC, respectively, after adjusting for age and sex (P for trend < 0.0001). Similar results were observed in the multivariate adjusted model, with ORs of 1.28 (95% CI: 1.07 - 1.52) and 2.16 (95% CI: 1.48 - 3.16) for the HbA1c levels between 5.7% and 6.4% group and HbA1c ≥ 6.5% group, respectively.

### 4.3. Dose-response Analysis of Glycated Hemoglobin with Coronary Artery Calcification

**Figure 2.**
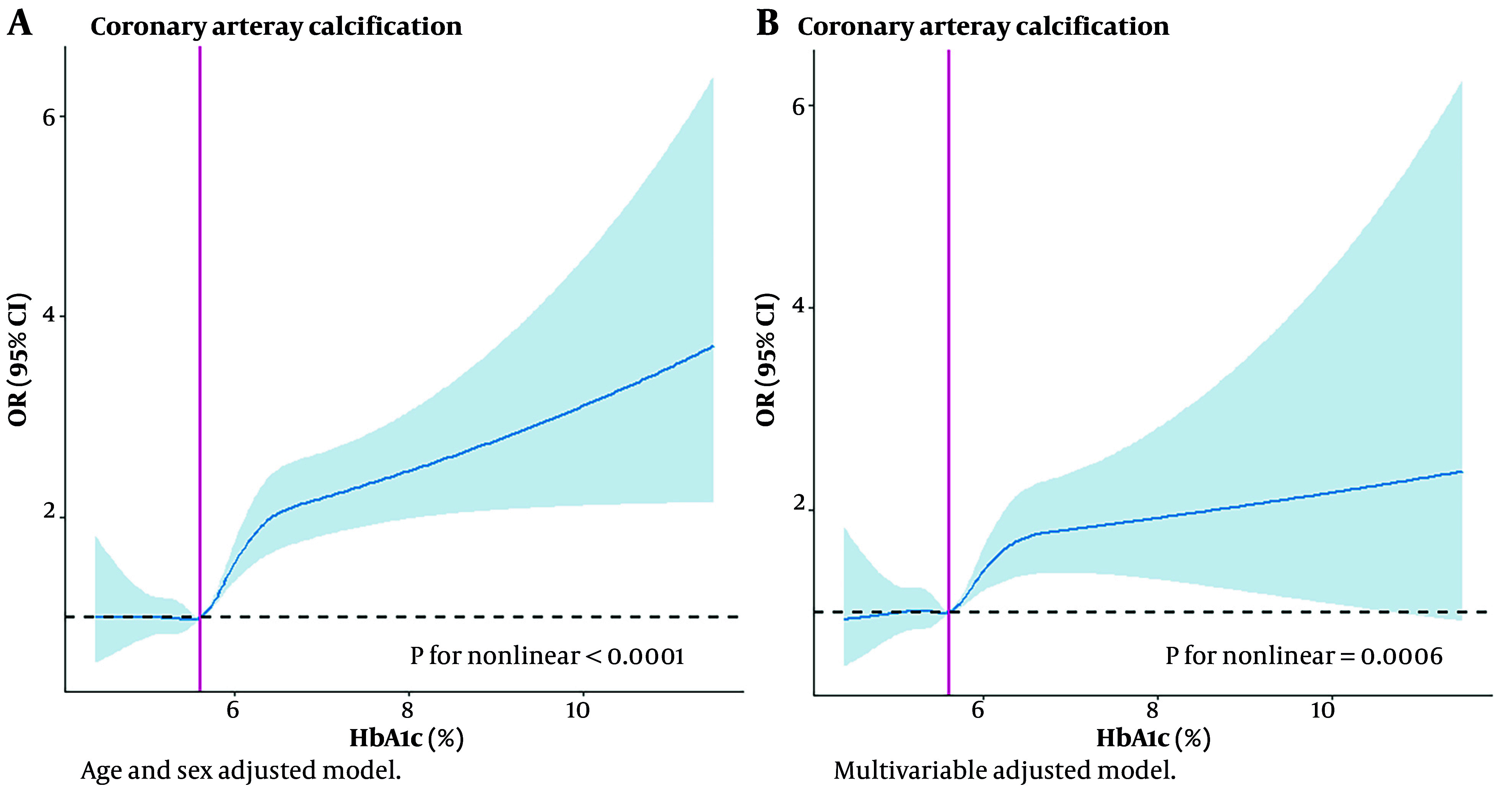
Exposure-response curves for the association between glycated hemoglobin (HbA1c) and prevalence coronary artery calcification (CAC). A, adjusted for age and sex; B, adjusted for age, sex, Body Mass Index (BMI), systolic blood pressure, diastolic blood pressure, total cholesterol (TC), triglyceride (TG), LDL-cholesterol, HDL-cholesterol, uric acid (UA), estimated glomerular filtration rate (eGFR), fasting blood glucose (FBG) and 2h plasma glucose. The shading indicates 95% CIs. Abbreviation: OR, odds ratio.

Figure 2A demonstrated a non-linear association between HbA1c and CAC, with adjustments made for age and sex (P for nonlinearity < 0.0001). The exposure dose-response curves for HbA1c levels above 5.7% exhibited an upward-sloping trend. This trend was also evident in Figure 2B, which showed similar upward trends in a model adjusted for multiple variables.

### 4.4. Subgroup Analysis

**Figure 3.**
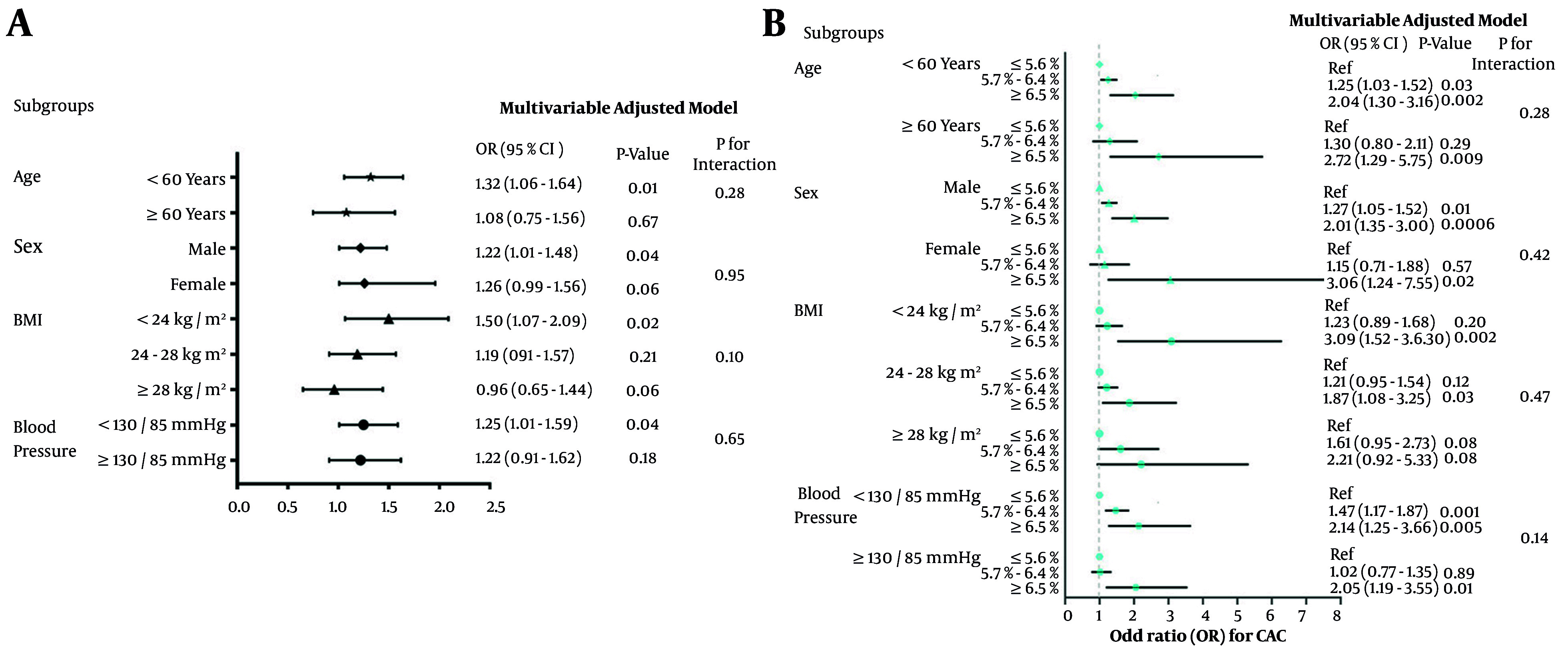
Association of glycated hemoglobin (HbA1c) with risk of coronary artery calcification (CAC) in subgroup analysis; multivariable adjusted model including age, sex, Body Mass Index (BMI), systolic blood pressure, diastolic blood pressure, total cholesterol (TC), triglyceride (TG), LDL-cholesterol, HDL-cholesterol, uric acid (UA), estimated glomerular filtration rate (eGFR), fasting blood glucose (FBG) and 2h plasma glucose. Abbreviation: OR, odds ratio.

The subgroup analyses in Figure 3 were conducted based on age (≥ 60 years and < 60 years), sex, BMI (normal, overweight, and obese), and blood pressure (normal and elevated). As shown in Figure 3A, after adjusting for age, sex, BMI, blood pressure, serum lipid, eGFR, UA, fasting plasma glucose, and 2-hour postprandial blood glucose, it was observed that the association between HbA1c and CAC was more pronounced in those aged less than 60 years, with normal weight and blood pressure less than 135/85 mmHg, although none of the interactions between HbA1c and subgroups were statistically significant. As shown in Figure 3B, individuals with HbA1c levels greater than 6.5% had a significantly increased risk of CAC across nearly all subgroups.

## 5. Discussion

Among a large population of 8,955 middle-aged and elderly Chinese individuals undergoing routine health screenings, it was noted that HbA1c levels were significantly linked to the risk of CAC. The study's key finding was that CAC was present in 18.99% of the participants, and the risk of CAC rose significantly with higher HbA1c levels. Studying HbA1c is crucial for several reasons. Firstly, it can help identify individuals who are at risk of developing type 2 diabetes mellitus (T2DM) (12). Secondly, elevated levels of HbA1c can signal a heightened risk of both subclinical and clinical cardiovascular disease, even in those who have not yet received a diabetes diagnosis (13). Lastly, monitoring HbA1c levels can result in interventions that may help prevent the onset of T2DM, subclinical atherosclerosis, and cardiovascular disease (14). Glycated hemoglobin serves as a dependable indicator of blood glucose levels for a span of 2 to 3 months, and if it measures 6.5% or higher, it is indicative of a T2DM diagnosis (15). However, this definition mainly focuses on the association between plasma glucose levels and microvascular complications of diabetes, rather than macrovascular complications (16). Recent meta-analyses indicate that individuals with prediabetes, characterized by slightly elevated FBG or HbA1c levels, are at a greater risk of developing cardiovascular disease (17). Moreover, a study conducted over a prolonged period has shown that the likelihood of developing coronary heart disease and stroke can begin at HbA1c levels lower than those typically used to diagnose T2DM (18). This research study has confirmed that HbA1c is an independent predictor of CAC risk, even after accounting for traditional risk factors and levels of fasting and postprandial glucose. The risk of developing CAC was found to increase not only when HbA1c levels were above the diabetes diagnostic threshold of 6.5%, but also when levels were between 5.6% and 6.4%. The relationship between HbA1c levels and CAC risk was shown to be dose-dependent, with higher levels of HbA1c leading to a higher risk of CAC. The HbA1c is a reliable marker of long-term glucose exposure, including postprandial spikes, and is more stable than fasting glucose levels. While HbA1c may contribute to vascular damage, it primarily reflects the presence of other glycated molecules involved in vascular inflammation and atherosclerosis (19). Because of its reliability, widespread availability, and cost-effectiveness, HbA1c measurement can serve as a useful tool for evaluating subclinical atherosclerosis and the risk of cardiovascular disease in individuals who do not meet the criteria for T2DM, without requiring fasting samples.

The results of our subgroup analyses indicated that individuals under 60 years old, with normal weight, and with blood pressure below 130/85 mmHg who had elevated HbA1c levels were at a significantly higher risk of developing CAC. This phenomenon may be attributable to the observation that individuals of younger age, with normal BMI and blood pressure, tend to exhibit a lower prevalence of cardiovascular risk factors. Consequently, the influence of elevated HbA1c levels on atherosclerosis becomes more pronounced. This implies the need for early intervention to decrease HbA1c levels or consider more aggressive management of CVD risk factors in this subgroup. There is compelling evidence that primary prevention is effective in delaying the progression from prediabetes to type 2 diabetes, which is expected to enhance outcomes (19). Lifestyle interventions, such as making dietary changes, increasing physical activity, and managing weight, are already recommended for individuals with prediabetes (class IA) (20). Additionally, certain diabetes medications like metformin, GLP-1 inhibitors, and SGLT2 inhibitors can also be used by individuals without diabetes (20). Managing blood glucose levels and decreasing HbA1c levels is critical for those with significantly elevated HbA1c levels and a high risk of CAC to prevent cardiovascular events. Detecting cardiovascular and cerebrovascular diseases early is essential for decreasing the occurrence of major cardiovascular adverse events and enhancing prognosis. Coronary atherosclerotic heart disease is a prevalent cardiovascular condition, and CAC serves as an early independent predictor (21). CT scans have high sensitivity in detecting CAC, surpassing other imaging methods in detection rate. Electrocardiogram-gated conventional dose CT plain scans are commonly used for noninvasive screening of CAC, identifying both clinical and subclinical coronary atherosclerosis (22). In recent years, low-dose chest CT (LDCT) has become a popular method for lung cancer screening, recommended by the International Early Lung Cancer Action Program and National Lung Screening Trial Research (23). Some researchers have successfully used non-electrocardiogram-gated conventional dose CT plain scans to identify and quantify CAC, with non-electrocardiogram-gated LDCT showing good agreement with conventional dose CT scans (24, 25). Using LDCT as an initial screening method for coronary heart disease shows promise, as it enables simultaneous early detection of lung cancer and CAC. This strategy is both cost-effective and time-efficient, while also minimizing radiation exposure for patients, making it ideal for regular health check-ups.

The study used a large sample size from a group of people undergoing health checkups and accounted for factors like gender, age, BMI, blood pressure, lipid levels, UA, and plasma glucose to enhance the accuracy of the findings. However, there are some limitations to consider. Firstly, the link between HbA1c and CAC cannot be assumed as causal due to the observational nature of the study. Secondly, the study only included individuals receiving healthy physical exams, which may restrict the applicability of the results to the general population. Thirdly, there may have been residual confounding from both unmeasured and measured variables, despite attempts to adjust for known risk factors through multivariate modeling. Fourthly, HbA1c measurements can be affected by factors such as hemoglobin variants, genetic hemoglobinopathies, thalassemia, and iron deficiency anemia. Nevertheless, HbA1c remains a valuable marker of long-term glycemic control with less variability compared to impaired glucose tolerance and impaired fasting glucose measurements. Finally, in this study, we did not measure the CAC score but made a qualitative diagnosis by a radiologist from a chest CT scan. Therefore, we cannot provide a continuous correlation analysis. In future studies, we will consider including the CAC score to more accurately analyze its association with HbA1c.

In conclusion, we have found a connection between HbA1c levels and CAC. Individuals with mildly elevated HbA1c levels and those with levels indicating type 2 diabetes had a higher prevalence and increased risk of CAC. This link supports existing evidence that diabetes and prediabetes increase the risk of subclinical cardiovascular disease. Our study suggests that maintaining stable blood glucose levels to keep HbA1c below 5.7% could serve as a potential target for interventions to prevent the progression from subclinical disease to clinical events. Further prospective research is needed to confirm this association, and randomized controlled trials are necessary to investigate the potential value of reducing HbA1c in preventing subclinical cardiovascular disease.

ijem-23-1-158710-s001.pdf

## Data Availability

The datasets generated and analyzed during the current study are not publicly available but are available from the corresponding author on reasonable request.
