# Supplementary material for: Association Between Glycated Hemoglobin and Coronary Artery Calcification in Middle-Aged and Elderly Chinese Checkup Populations
Source: Int J Endocrinol Metab. 2025 Apr 21;23(1):e158710. doi: 10.5812/ijem-158710 (PMC12118369; doi:10.5812/ijem-158710)
Supplement: ijem-23-1-158710-s001.pdf [file ijem-23-1-158710-s001.pdf]

Appendix 1. Associations Between Glycated Hemoglobin and Coronary Artery Calcification in Middle-Aged and Elderly Chinese Checkup Populations

Table S1. Quality Control Data for the Laboratory Testing

Laboratory Indicators	Bias (%)	Intra-assay CV	Inter-assay CV
TC	(-3.76%) - (-1.10%)	2.3%	2.8%
TG	(-5.48%) - 2.76	4.4%	3.3%
HDL-C	(-9.71%) - (-1.54%)	4.1%	3.1%
LDL-C	(-3.45%) - (-0.53%)	4.2%	2.1%
FBG	(-2.38%) - 1.86%	2.8%	3.0%
UA	(-5.06%) - 1.58%	4.1%	3.2%
SCR	(-3.62%) - 2.78%	3.7%	2.8%
HbA1c	(-0.88%) - 2.66%	2.8%	1.9%

Abbreviations: FBG, fasting blood glucose; TG, triglyceride; TC, total cholesterol; HDL-C, high density lipoprotein cholesterol; LDL-C, low density lipoprotein cholesterol; SCR, serum creatinine; HbA1c, glycated hemoglobin.
